# Predicting and comparing three corrective techniques for sagittal craniosynostosis

**DOI:** 10.1038/s41598-021-00642-7

**Published:** 2021-10-27

**Authors:** Connor Cross, Roman H. Khonsari, Dawid Larysz, David Johnson, Lars Kölby, Mehran Moazen

**Affiliations:** 1 Department of Mechanical Engineering, University College London, London, UK; 2 Department of Maxillofacial Surgery and Plastic Surgery, School of Medicine, Necker – Enfants Malades University Hospital, Assistance Publique – Hôpitaux de Paris, University of Paris, Paris, France; 3 Department of Head and Neck Surgery for Children and Adolescents, University of Warmia and Mazury in Olsztyn. Ul, Zolnierska 18a, 10-561 Olsztyn, Poland; 4 Oxford Craniofacial Unit, Oxford University Hospital, NHS Foundation Trust, Oxford, UK; 5 Department of Plastic Surgery, Sahlgrenska University Hospital, The Sahlgrenska Academy, University of Gothenburg, Gothenburg, Sweden

**Keywords:** Biomedical engineering, Mechanical engineering

## Abstract

Sagittal synostosis is the most occurring form of craniosynostosis, resulting in calvarial deformation and possible long-term neurocognitive deficits. Several surgical techniques have been developed to correct these issues. Debates as to the most optimal approach are still ongoing. Finite element method is a computational tool that’s shown to assist with the management of craniosynostosis. The aim of this study was to compare and predict the outcomes of three reconstruction methods for sagittal craniosynostosis. Here, a generic finite element model was developed based on a patient at 4 months of age and was virtually reconstructed under all three different techniques. Calvarial growth was simulated to predict the skull morphology and the impact of different reconstruction techniques on the brain growth up to 60 months of age. Predicted morphology was then compared with in vivo and literature data. Our results show a promising resemblance to morphological outcomes at follow up. Morphological characteristics between considered techniques were also captured in our predictions. Pressure outcomes across the brain highlight the potential impact that different techniques have on growth. This study lays the foundation for further investigation into additional reconstructive techniques for sagittal synostosis with the long-term vision of optimizing the management of craniosynostosis.

## Introduction

Sagittal craniosynostosis is the result of the premature fusion of the sagittal suture, with an occurrence rate of 1 in every 10,000 live births^1–4^. It is the most common form of craniosynostosis, with several studies reporting a significant increase in its presents over the last 30 years^5,6^. Raised intracranial pressure, potentially leading to cognitive impairment has been related to the calvarial deformation^3,7,8^. The first corrective techniques were developed in the late nineteenth century to restore the normative skull shape^9,10^. In recent times, craniofacial centres have adopted a number of techniques. These range from strip craniotomy (removal of the fused suture) and total calvarial remodelling (reshaping of bone) to spring assisted cranioplasty (bone widening using springs) and helmet therapy (postoperative skull shaping)^11–15^. As a result, the most optimum method of treatment and their respective outcomes are still debated among craniofacial surgeons^16–20^.

Finite element (FE) method is a powerful computational tool used to analyse a wide range of engineering solutions^21^. Recently, FE studies have investigated the management of craniosynostosis^22–26^. Advanced methods have accurately simulated calvarial growth and bone formation in developed models^27–32^. Such methods have the potential to investigate the biomechanics of craniosynostosis and predict various sagittal synostosis outcomes under a range of reconstructions. However, validating our approach with pre-existing data is critical for building confidence in our FE predictive results^33^.

The aim of this study was to investigate the potential biomechanical differences between three corrective techniques used for the management of sagittal craniosynostosis i.e. two variations of spring-assisted cranioplasty (SAC) vs. modified strip craniotomy (MSC) using a generic FE approach. The primary intention for this research was to directly compare the spring vs. the strip techniques since from a biomechanical point of view the main difference between these techniques are the width of craniotomy and the presence or absence of the springs.

## Materials and methods

A preoperative generic 3D model of a sagittal craniosynostosis patient at 4 months of age was developed based on computed tomography (CT) data. This generic model was then virtually reconstructed based on two variations of spring-assisted cranioplasty (SAC) and the modified strip craniotomy (MSC). Post-operative calvarial growth was modelled using the FE method. Given the importance of validation of the computational models, results obtained from the SAC methods were compared versus a series of in vivo CT data while results obtained from the MSC technique were compared vs. published data in the literature. The overall morphology of the skull, spring displacement, the pattern of bone formation across the calvarial, and the level of contact pressure that each technique imposes on the growing brain (here, the intracranial volume) was investigated post-operatively. Note the generic preoperative model used in this study was described and validated in detail elsewhere^34^.

### Surgical techniques

**Figure 1:**
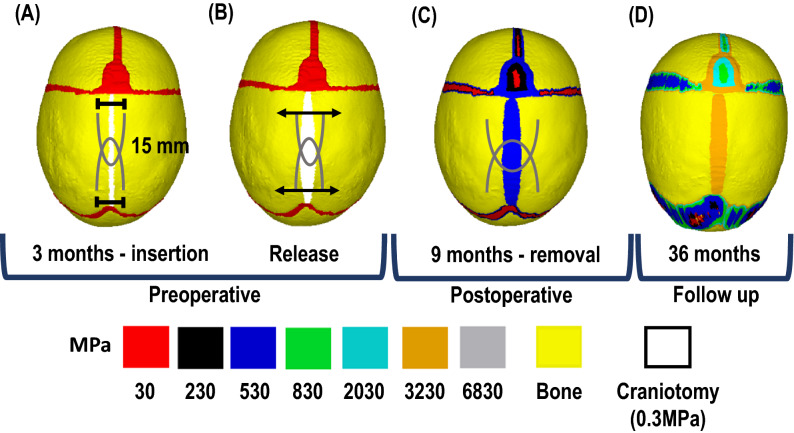
Simulation workflow. All techniques were replicated at 4 months of age, when spring insertion (**A**) and ‘release’ (**B)** were replicated for SAC. Skull growth, calvarial healing and bone formation at sutures were replicated up to 9 months of age, when springs were removed (**C**). Skull growth then continued up to 36 months of age [D].

*Spring-assisted cranioplasty (SAC):* The SAC procedure and parameters replicated in this study were based on the standard Gothenburg procedure, as detailed by Lauritzen et al.,^10^ and more recently by Satanin et al.,^35^. A 1 mm wide craniotomy, extending from the anterior fontanelle to lambdoid suture was performed. Two holes were burred approximately 15 mm apart, across the craniotomy for spring placement (Fig. 1A). These were performed 40 mm (anterior spring), 55 mm (middle spring – for 3 SAC) and 75 mm (posterior spring) from the coronal suture. The quantity of springs used can vary between two (i.e. 2 SAC) and three (i.e. 3 SAC). In situ spring displacement of approximately 5 mm occurs naturally (denoted as: ‘release’), allowing for mediolateral widening upon insertion of the springs (Fig. 1B). These were then removed in a secondary procedure 5 months post-insertion (Fig. 1C). After which, calvarial growth continued unaided (Fig. 1D).

*Modified strip craniotomy (MSC):* For our comparative technique, we reconstructed the procedure described by Thomas et al.,^13^. In brief, A 50 mm wide vertex craniotomy was created across the anteroposterior, extending from coronal to lambdoid.

### Image processing

A previously described model was used for this study^34^. In short, CT data of a pre-operative sagittal synostosis patient at 4 months of age was obtained from the Hôpital Necker – Enfants Malades Craniofacial Surgery Unit (Centre de Référence Maladies Rares Craniosténoses et Malformations Craniofaciales CRANIOST, Paris, France). Full ethical protocol for undertaking this study was approved by the institutional review board and committee from the Necker – Enfants Malades University Hospital. Informed consent was granted from the patient’s guardian. All patient information was anonymized prior to the retrieval of CT data in accordance with the HIPAA (1996). Image resolution was measured at 0.625 × 0.625 mm. Full consent was granted by the child’s guardians for the purposes of this study. The image processing package, Avizo (V9.2.0; Thermo Fisher Scientific, Mass, USA) was used for 3D model development. The calvarial bone, sutures, and intracranial volume (ICV i.e. all internal calvarial components) were all segmented in preparation for FE simulations. Calvarial bone was automatically highlighted using the Hounsfield scale method. Sutures and ICV were highlighted manually. The detailed 2 SAC, 3 SAC and MSC craniotomies, based on the techniques described above, were then replicated on the pre-operative model prior to calvarial growth.

### Finite element analysis

A quadratic tetrahedral mesh consisting of 4 million elements in total was selected after a mesh convergence study. Where 3,100,000 elements were used to mesh the bone, sutures, and craniotomy based on the von Mises strain and 900,000 elements were used to mesh the ICV based on the contact pressure. Mesh convergences was seen to have been achieved once both the strain and pressure values had plateaued by ± 5%. Alterations to individual element geometries were performed to reduce the initial penetration between elements and decrease the aspect ratio. The fully meshed model was then imported into the FE package, ANSYS (V19.0; Canonsburg, PA, USA), to simulate calvarial growth, bone formation and contact between the ICV-inner calvarial interface. All materials were defined as linear isotropic. Bone, ICV, suture and craniotomy properties were assigned an elastic modulus of 421 MPa, 10 MPa, 30 MPa and 0.3 MPa, respectively^32,34,36,37^. Sensitivity tests were carried out which varied these stiffnesses initially (see: Supplementary Table S1 & S2) to achieve the target craniotomy widening (i.e. approx. 5 mm) seen after spring ‘release’. Both the ICV and craniotomy Poisson’s ratio was selected as 0.1. A Poisson ratio of 0.3 was selected for the bone and sutures.

*Boundary conditions:* A Hertzian frictional contact method was used to predict pressure changes across the ICV-inner calvarial interfaces, as previously implemented by Malde et al.,^32^. To summarise, a penalty-based surface to surface contact was established with a normal contact stiffness of 50 N/mm, a penetration tolerance of 0.5 mm and a normal/tangential friction coefficient of 0.1 to reduce the level of penetration. These surfaces were initially in contact, which then allowed the freedom of movement in the normal/tangential direction during skull growth. All bone-suture, bone-craniotomy and craniotomy-suture interfaces were assumed to be in bonded contact, with no relative motion or separation authorised. Nodal constraints were placed around the foramen magnum and across the nasal ridge in all degrees of freedom to avoid rigid body motion. Thermal expansion analogy was used to model the ICV growth as previously described by Libby et al.,^31^. Here, the ICV was increased from the initial pre-operative volume (measuring 659 ml) to the target in vivo follow-up volume in five load-steps for both SAC (i.e.1240 ml) and six for MSC (i.e. 1376 ml). The predicted target volumes were correlated with values seen in the literature to estimate the age of the model at each load-step^38^.

*Bone formation:* A previously described algorithm detailed by Marghoub et al.,^28^ was implemented to simulate the bone formation at the sutures and craniotomies during calvarial growth. In brief, elements were selected at a specified radius along the bone-suture/bone-craniotomy linings. The elastic modulus of these newly and previously selected elements was increased by 100 MPa for each month of growth. The elastic modulus of bone was also increased by 125 MPa for each month of growth. These changes in the elastic modulus of the bone/newly formed bone were estimated based on extrapolation of the bone properties that were measured during the development of normal mouse^37^ to human (considering ICV growth). A radius of 0.2 mm for every month of calvarial growth was selected for the coronal, lambdoid and squamosal suture formation based on observations in literature^39,40^ and prior sensitivity studies^34^ to predict the timing of closure. The metopic suture and anterior fontanelle were set to completely form by 24 months of age to represent the in vivo scenario^41,42^. A sensitivity study was carried out to investigate the morphological effect of different rates for bone formation at the craniotomies (see: Supplementary Table S3 & Figure S1). Following these sensitivity tests, a rate of 10.8 mm per month of growth was specified for the rate of calvarial healing. After each load-step, the geometry of the skull, displacement across the springs length and forces were updated to the newly deformed shape and values, respectively, which was then used to estimate the morphology of the skull at the next step/age. No adaptive remeshing algorithm was used here, as the geometry was updated at each interval. This approach avoided element distortions that would have otherwise occurred due to the large deformations occurring.

*Spring mechanics:* To replicate the characteristics of the SAC, linear spring elements (i.e., COMBIN14) were positioned approximately 15 mm across the craniotomy at insertion (i.e. 5 mm from the craniotomy into the parietal bone on either side plus the 5 mm gap between equalling to a total of 15 mm – see Fig. 1A). These elements behave under Hookean law, where the outward force was directly proportional to the level of tension/compression^26,43^. Here, a series of in vitro measurements were carried out to identify the force–length relationship of the springs (See Appendix S6). In short, an average force of 8 N was produced when crimping a wire initially measuring 100 mm to 15 mm (based on leg-to-leg measurements – See: Fig. 1A). These values were used to calculate the spring stiffness (K) at ‘release’ using Eq. 1:

1 $$K = f/dx$$

where $f$ represents the bilateral force and $dx$ represents the change in spring displacement (here initially, 100 mm minus 15 mm). A sensitivity test was carried out to investigate the effect of altering the initial spring force values by updating the spring stiffness on the predicted morphology (see: Supplementary Table S4 & S5). During ‘release’ and calvarial growth, spring forces and spring leg distances values were automatically calculated and updated using Eq. 2:

2 $$f = K*dx$$

Upon removal, the modelled springs were given a fixed force of 0 N. The growth then continued unaided to the target follow up age. Note that the spring stiffness remained unchanged throughout all simulations.

*Simulation and measurements:* Both SAC and MSC techniques underwent calvarial growth up to the follow-up ages of 36 and 60 months, respectively. Both predicted SAC calvarial morphologies were compared against a series of patient CT data sets undergoing the standard Gothenburg SAC procedure and retrieved from the Department of Plastic Surgery at the Sahlgrenska University Hospital (Gothenburg, Sweden). Full ethical protocols for undertaking this study were reviewed and approved by the institutional review board and committee at the Department of Plastic Surgery at the Sahlgrenska University Hospital. Informed consent was granted from all patient’s guardians. All patient CT information provided was anonymized in accordance with the Health Insurance Portability and Accountability Act of 1996 (HIPAA). CT data was grouped in accordance with the number of springs used for the treatment and classified as 2 SAC (n = 10) and 3 SAC (n = 8), respectively. The pre-operative CT for both groups were taken at a mean age of 4.9 ± 1.3 and 4.1 ± 0.7 months, respectively. Post-operative CT was taken at 10 ± 1.3 months of age, where the springs were removed. Follow-up CT was taken at 36 ± 2.0 months of age. Predicted MSC morphology was compared against reported CI outcomes of the same technique detailed by Thomas et al.,^13^ as CT data for this technique was unavailable for direct morphological comparisons. Measurements of the length (from glabella to opisthocranion), width (between the left and right euryons) and circumference were undertaken. The cephalic index (CI) was calculated by multiplying the width against the length and dividing by one hundred. 3D distance mapping was also used to observe predicted under- or over-estimation vs. the CT data provided. Our predicted morphology was compared against a single CT skull that matched closest to the overall mean length/width measurements within both SAC groups. The predicted spring opening was measured during skull growth and compared against CT data at 9 months of age by manually measuring the leg-to-leg distance against each CT patient data using the aforementioned image processing software. Predictive bone formation was recorded throughout our simulations to observe differences in suture and craniotomy closure times between techniques. Contact pressure across the ICV surface was recorded to observe the effects each considered technique had on the brain (here ICV) growth.

## Results

### Morphological comparisons

Table 1 provides a summary of the in vivo CT and predicted measurements corresponding to each technique at different ages. At pre-operative, the 4 months of age model used for the FE simulations measured a skull length, width, circumference, ICV and cephalic index of 137.2 mm, 108.1 mm, 430.6 mm, 659.9 ml and 78.7, respectively. The average age of patients who were treated with 2 SAC, 3 SAC (from our CT data) and MSC (from the literature^13^) were 4.9 ± 1.3, 4.1 ± 0.7 and 6 months (range: 3.1–9.5), respectively with corresponding CI of 76.9 ± 2.7, 74.3 ± 3 and 65.7 ± 4.7.

**Table 1:** Overview of predicted vs. in vivo measurements across all technique. Dashes indicate unavailable data.

	2 SAC		3 SAC		MSC [Thomas et al., 2015]		
	Clinical data	Prediction data	Clinical data	Prediction data	Clinical data	Prediction data	
n:	10	1	8	1	34	1	
(%) Male:	80	1	50	1	N/A	1	
Preoperative							
Age (months):	4.9 ± 1.3	N/A	4.1 ± 0.7	N/A	6.0 ± 3.1–9.5	N/A	
Mean length (mm):	148.5 ± 6.1	N/A	150.5 ± 9.9	N/A	-	N/A	
Mean width (mm):	114.3 ± 5.7	N/A	111.5 ± 5.6	N/A	-	N/A	
Mean circumference (mm):	455.3 ± 68.0	N/A	457.2 ± 27	N/A	-	N/A	
Mean intracranial volume (ml):	800.9 ± 102.1	N/A	800.8 ± 88.6	N/A	-	N/A	
Mean cephalic index:	76.9 ± 2.7	N/A	74 ± 3.4	N/A	65.7 ± 4.7	N/A	
Postoperative:							
Age (months):	10.9 ± 1.3	9.0	10.6 ± 0.3	9.0	12	9.0	12.0
Mean length (mm):	162.5 ± 8.0	143.3	165.2 ± 6.1	142.4	-	143.2	143.4
Mean width (mm):	129.8 ± 5.0	112.5	129.1 ± 6.6	113.6	-	112.9	116.2
Mean circumference (mm):	486.5 ± 59.4	397.3	429.0 ± 107.0	397.2	-	395.5	416.8
Mean intracranial volume (ml):	1089.2 ± 144.9	829.5	1131.2 ± 130.5	829.5	-	817.4	1007.0
Mean cephalic index:	79.9 ± 2.9	78.5	78.2 ± 4.5	79.7	73.3 ± 5.2	78.8	81.1
Follow up							
Age (months):	37.15 ± 2.0	36.0	37.6 ± 1.3	36.0	60	36	60
Mean length (mm):	176.9 ± 9.3	163.8	178.8 ± 8.8	163.4	-	155.8	155.1
Mean width (mm):	135.1 ± 5.4	122.3	132.7 ± 6.4	121.2	-	122.3	124.7
Mean circumference (mm):	512.4 ± 35.4	454.4	523.2 ± 37.0	453.3	-	429.4	437.0
Mean intracranial volume (ml):	1245.0 ± 166.8	1261.0	1239.0 ± 133.8	1261.0	-	1240.4	1376.9
Mean cephalic index:	76.4 ± 2.5	74.6	74.3 ± 3.8	74.1	71.5 ± 4.3	78.4	80.3

The pre-operative FE model used for this study was at the age of 4 months with skull length, width, circumference, ICV and cephalic index of 137.2 mm, 108.1 mm, 430.6 mm, 659.9 ml, 78.7 respectively. NA at the pre-operative stage for prediction data corresponds to the initial FE model that was the same model across all techniques that was then reconstructed to replicate each technique i.e. predicted the shape at different ages.

**Figure 2:**
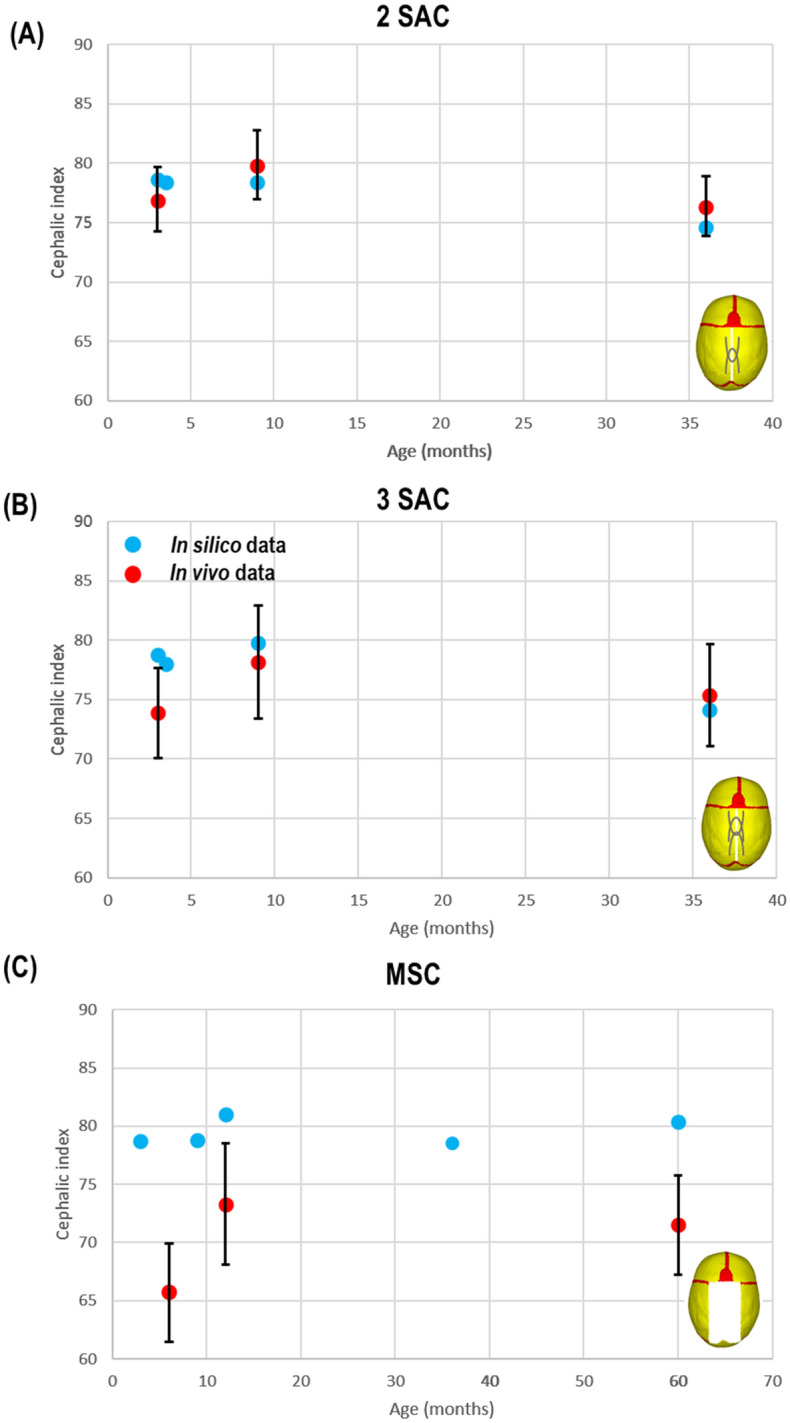
Predicted vs. in vivo cephalic index data with SD. Showing 2 SAC (**A**), 3 SAC (**B**) & MSC outcomes (**C**).

At the post-operative stage, the FE model predicted CI’s of 78.5, 79.7 and 78.8 at 9 months of age and 74.6, 74.1 and 80.3 at 36 months of age for the 2 SAC, 3 SAC and MSC technique, respectively. At 12 months of age, CI of 81.1 was predicted for MSC. The in vivo CT and literature^13^ CI measurements were 79.9 ± 2.9, 78.2 ± 4.5 and 73.3 ± 5.2, at 9–12 months of age, and 76.4 ± 2.5, 74.3 ± 3.8 and 71.5 ± 4.3 at 36 months of age for the 2 SAC, 3 SAC and at 60 months of age for the MSC, respectively. Hence, while the FE model captured the post-operative relapse in the SAC techniques, it failed to capture the relapse in the MSC technique (Fig. 2).

### Spring opening

**Figure 3:**
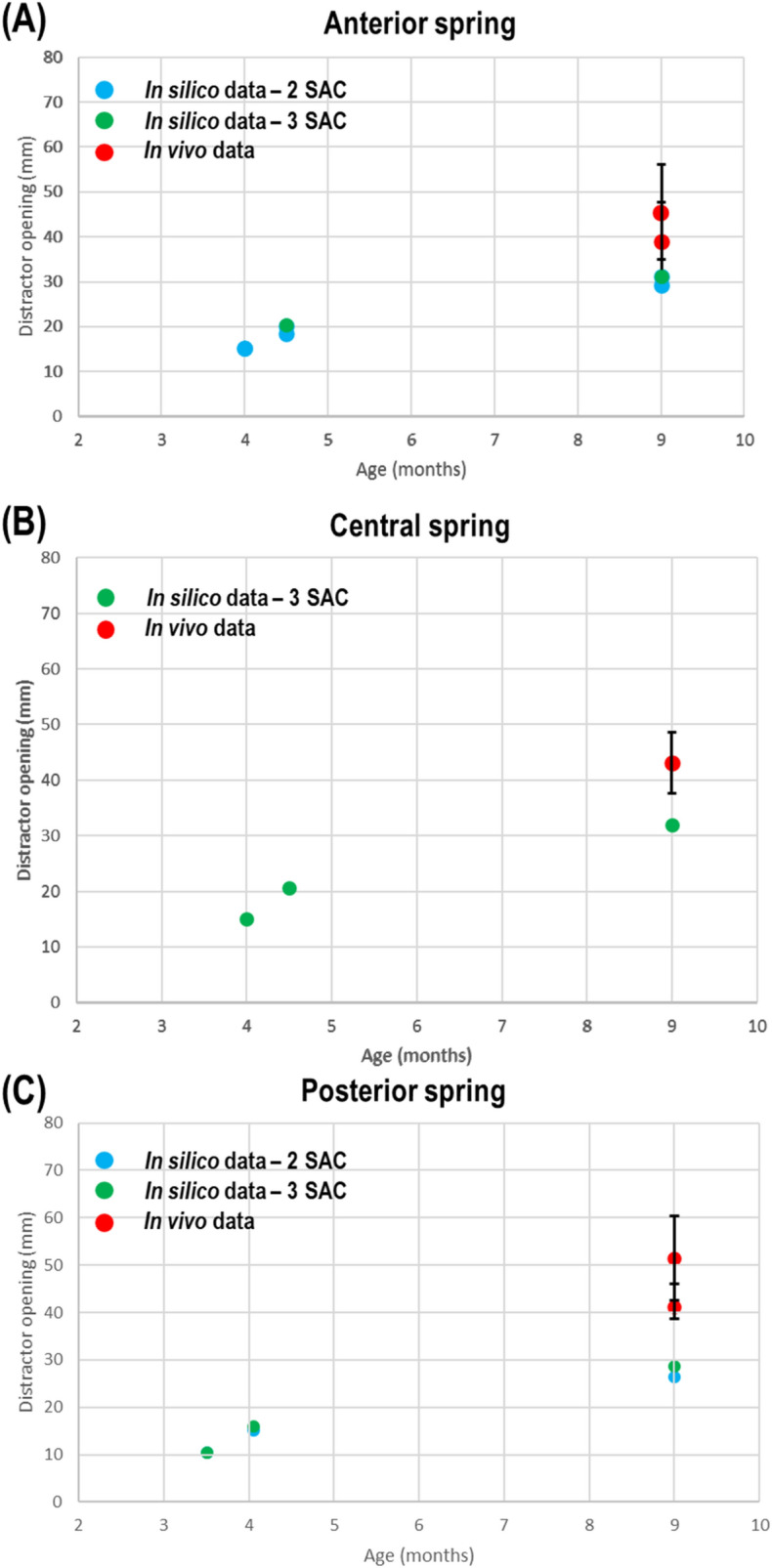
Predicted vs. in vivo spring opening data with SD. Showing anterior (**A**), central (**B**) & posterior (**C**) springs in both SAC techniques. Diagrams show regions where measurements were performed.

Increased spring opening from insertion (15.2 mm) to ‘release’ (19.5 mm) was predicted in both SAC techniques, which in turn lead to a 5 mm widening of the craniotomy (Fig. 3). By 9 months of age, FE models under-predicted the spring opening data observed in vivo in the anterior (29.2 mm; 31.1 mm vs. 45.5 ± 10.5 mm; 39.0 ± 8.5 mm), central (31.9 mm vs. 43.1 ± 5.0 mm) and posterior springs (29.2 mm; 31.3 mm vs. 51.4 ± 8.9 mm; 42.3 ± 3.7 mm).

**Figure 4:**
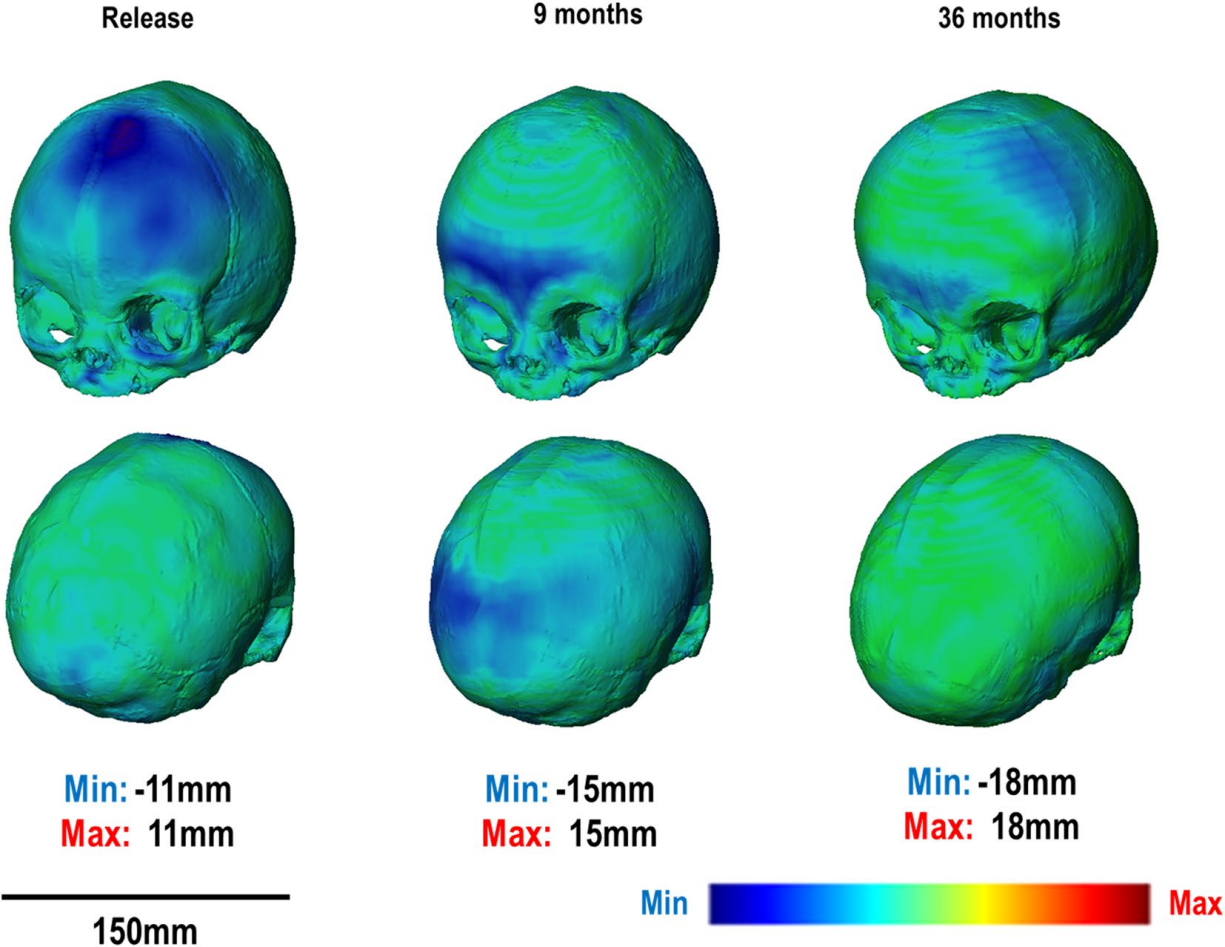
2 SAC 3D distance plot at respective ages against mean in vivo CT skull.

**Figure 5:**
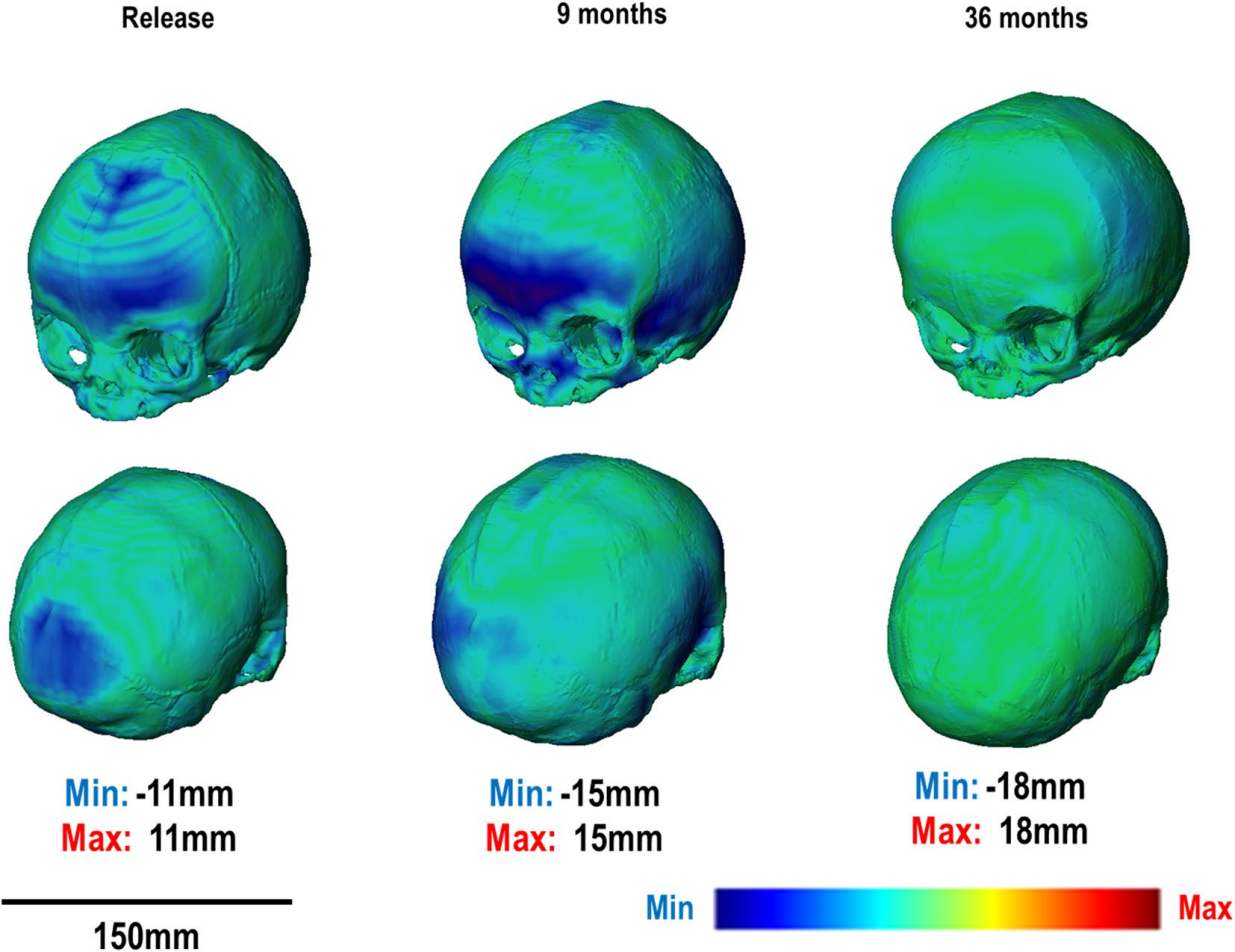
3 SAC 3D distance plot at respective ages against mean in vivo CT skull.

3D displacement mapping results highlight the under- and over-prediction at several ages for both 2 SAC and 3 SAC techniques (Fig. 4 & 5, respectively). Note that pre-operative CT data is compared against the predictive release morphology. An under-prediction of the anterior and posterior regions was evident from release to post-operative (i.e. 9 months) across both techniques. By follow up (i.e. 36 months), a good morphological match was observed, with minimal under-prediction across the mediolateral.

### Bone formation

**Figure 6:**
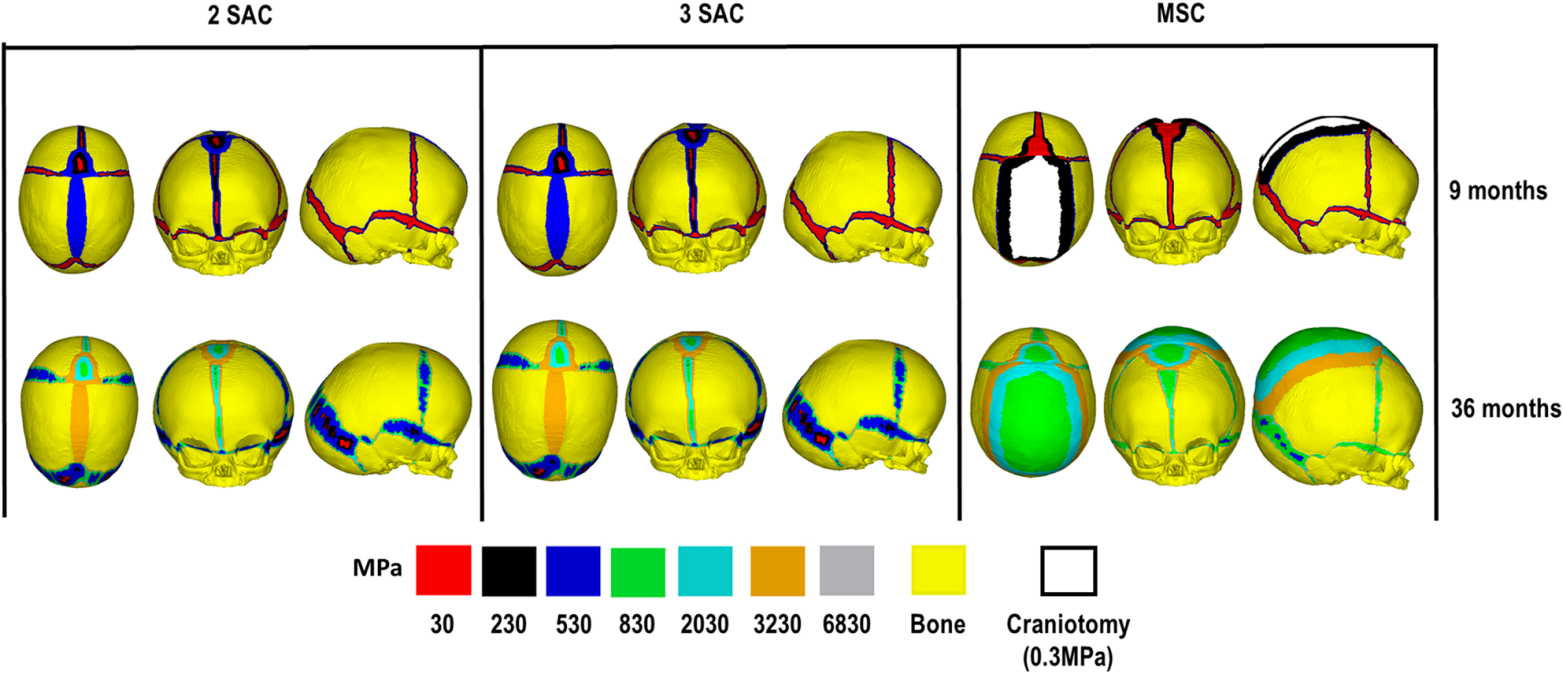
Bone formation predictions across sutures/craniotomy across all techniques.

Predicted bone formations and overall calvarial morphologies are shown in Fig. 6. At 9 months of age, both SAC techniques predicted complete closure of the craniotomy, while MSC showed large areas of patency. All sutures showed little formation by this age. By 36 months of age, bone was formed across all the sutures in all considered techniques, with some patency observed at the lambdoid suture in the SAC method. By this time, new bone was formed at the MSC craniotomy, with all sutures showing complete closure and narrowing compared to the SAC outcomes. Comparing the overall predicted morphology of the skull at 36 months of age between both SAC and MSC techniques highlighted the larger anteroposterior growth of the skull in the SAC technique in contrast to the larger dorsoventral growth of the skull in the MSC technique.

### Contact pressure

**Figure 7:**
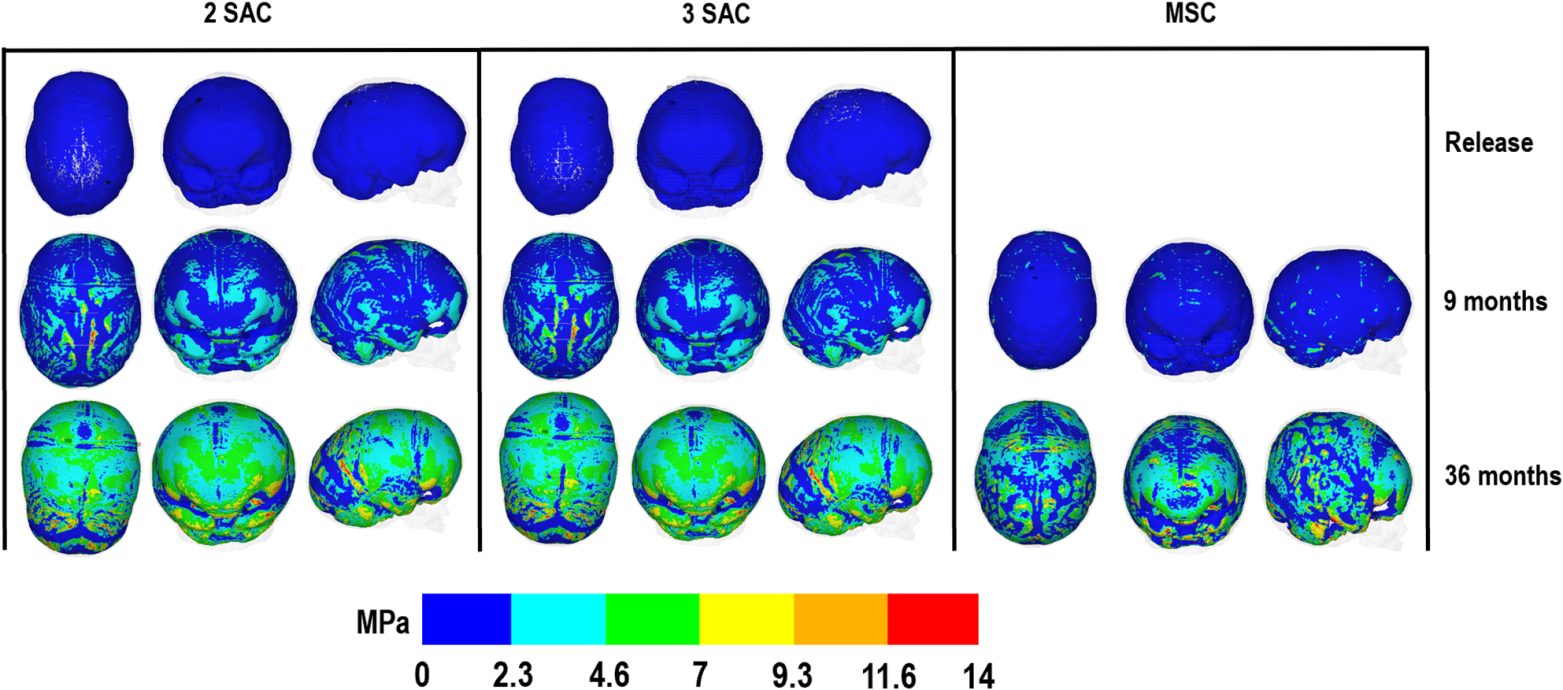
ICV pressure predictions across ICV-bone surface for all techniques.

Brain growth and contact pressure across the ICV at different ages are shown in Fig. 7. When simulating spring release, pressure changes were negligible. At 9 months of age, greater pressure was observed across the ICV in both SAC vs. MSC. At 36 months of age, an even distribution of the pressure was observed in the SAC vs. MSC. Greater concentration of high pressure was observed at the anterior, mediolateral and across the anterior fontanelle in MSC while both SAC techniques highlighted minor elevated levels of pressure at the mediolateral sides of the skull in the temporal regions.

## Discussion

Many variations of sagittal craniosynostosis correction exist, ranging from invasive to non-invasive procedures^14^. Large debates over the optimal outcome between techniques are still ongoing. Since the mid-twentieth century, computational models using finite element (FE) method have been widely used to investigate the biomechanics of a range of clinical conditions and their managements^44–46^. FE shows promise in assisting with the management of various forms of craniosynostosis^33^. In this study, we attempted to illustrate the use of FE method in which the biomechanics of three corrective techniques were compared. Morphological outcomes were compared against our own CT data used for this study and literature data at various postoperative and follow up time points. Our results highlight the potential impact of the surgical techniques on the overall morphology of the skull, the pattern of bone formation across the craniotomies and other sutures as well as the pressures that they may apply across the whole intracranial volume. The work here shows promising perspectives in optimizing the management of craniosynostosis.

### Morphological comparisons

Our results under-predicted changes in skull length and width in predictive vs. in vivo data. This could have been attributed to predicted ICV measurements, particularly at postoperative time points. The simulations were run by increasing the ICV to an ‘average’ value at a specific age based on the literature and our previous studies^34,38^. However, when comparing the FE results vs. the average ICV of the patients considered in this study at 9–12 months of age, there was a 20% difference between the two (based on the SAC technique). This could explain the large under-predictions in morphological outcomes at this age range. A closer match was achieved at 36 months, as this variation (between the in silico and in vivo ICVs) was seen to reduce to 1% (based on the SAC technique). This closer match in volume by 36 months may be attributed to the reduction in growth seen after the first year of life vs. our predicted linear growth in this study. One could argue that the preoperative CT data from our SAC cohort could have been used to develop a FE model for a true validation of the FE results. However, we considered (1) using a generic model to compare different surgical techniques (2) to keep a level of consistency regarding the preoperative morphology between our compared techniques shown here and thus, chose to utilize our previously validated FE model^34^. The CI was seen to vary slightly from the preoperative period to the time of spring removal in predictive and CT data (Fig. 2). By 36 months, there was a reasonable match between the in silico and in vivo data. Although a more significant relapse was seen in predictive outcomes, it is interesting to see this postoperative pattern being accurately predicted. Further, the antero-posterior growth vector of the skull observed post-operatively in the SAC technique in vivo was also captured by the in silico FE results (Table 1).

Reported data for MSC by Thomas et al.,^13^ was limited for the present study. Nevertheless, a comparison of CI was undertaken to highlight the potentials as well as the limitations of our modelling approach. Greater changes were seen in reported in vivo data vs. our predicted data. Further, our predicted CI at 60 months of age overpredicted what was clinically observed in the study^13^ (see Fig. 2C). The differences between the in vivo and in silico results here could be due to a number of factors e.g. (1) the initial CI of the patient that we used to develop the FE models was considerably higher than the average pre-operative CI of the patients considered in the study of Thomas et al.,^13^ (i.e. 78.7 vs. 65.7). The pre-operative CI has indeed been shown to be clinically a major factor in determining the postoperative outcomes^13^; (2) there could have been minor surgical technical details that have not been captured in the simulations performed here; (3) It is also possible that the ICV of the patients in the study of Thomas et al.,^13^ were lower than the ‘average’ values that were used in the FE simulations to model skull growth in the present study. Nevertheless, we believe that the virtual comparative nature of the assessments made between the different techniques considered here is interesting and valuable. Allowing our predictions to determine the growth under two extreme conditions (i.e. 5 mm vs. 50 mm craniotomy). Our predictions, considering their limitations, highlights that SAC technique can perhaps lead to a more antero-posterior growth of the skull whereas MSC technique used here can perhaps lead to a more dorsal–ventral growth of the skull. All techniques demonstrated an improvement in the CI before relapsing, although this was seen to be greater in the MSC predictions. This difference was attributed to the greater increase in length seen in SAC vs. MSC and a reduction in width. It could be argued that if further growth was undertaken beyond 60 months for MSC, this relapse would continue beyond the value seen in the SAC. Considering morphological measurements shown, our current analysis highlights improved outcomes in the MSC vs. SAC predictions. On the other hand, it must be noted that the MSC technique is no longer performed at the Oxford Craniofacial Unit given that the study of Thomas et al.,^13,47^ highlighted that the total calvarial remodelling technique performed in this unit resulted in higher CI and better clinical outcomes for these patients.

### Spring opening

Considering both SAC techniques, although our comparison of spring opening distance was restricted to a single time point, predictive results appeared to match in the low range of in vivo data at 9 months (Fig. 3). However, reports from Windh et al.,^48^ and Lauritzen et al.^49^, agree well with the distance measured upon release. Our spring predictions only gain an additional 11 mm in length from release to 9 months. Other centres have documented these changes in greater detail. Yang et al.,^43^ studied the spring opening and bi-temporal displacement of SAC patients during the entire 3 months of treatment. Spring opening was seen to increase rapidly from 7–10 mm to 23 mm in the first 2 h after insertion. This rate of opening was seen to decrease to 4 mm after only 8 h following insertion, after which the length was seen to plateau. Although these larger displacements were not seen in our predictions, it should be noted that a larger spring forces were used upon insertion (14 N vs. 8 N). Further, such levels of in situ craniotomy and spring widening observed in this work do not fully reflect the larger levels seen in other craniofacial centres under different operative parameters^26^. However, as our intention was to focus on a single centres SAC conditions (i.e. Gothenburg, Sweden), such considerations were examined in a sensitivity analysis (See: Supplementary Table S4). Both 2 SAC and 3 SAC techniques show little change in opening spring length by 9 months, with all springs displacing by approximately 10 mm from ‘release’ to removal. Interestingly, incorporation of a middle spring for 3 SAC showed little effect on morphological outcomes, particularly that of biparietal widening. Nonetheless, these predictions, cross-referenced with our morphological measurements, may prove informative for surgeons in reducing the risk of damaging the sagittal sinus and/or lower risk of spring dislodgement as fewer distractors may be necessary to achieve the same morphological goals with regards to this study^50,51^.

### Bone formation

A previously developed approach to model bone formation detailed by Marghoub et al.,^28^ was adopted in this study. Given that the formation rate at the cranial sutures and craniotomies in humans could be different from what was used in our previous study, various sensitivity tests were carried out to justify the choice of this parameter (see Supplement Figure S1 & Table S3). Overall, we observed that the patterns of bone formation at different sutures and craniotomies appeared to match that of the in vivo observations from the literature^39–42^ and the CT cohort used at 9 and 36 months of age. For example, our results showed a greater posterior/occipital narrowing at 36 months of age in both SAC models. Such a phenomenon was caused by the fusion of the craniotomy and the patency of the lambdoid sutures, allowing for angular changes across the parietal bone plates, a phenomenon also reported in the clinical study of Satanin et al.,^35^. Further, considering the pattern of bone formation across the MSC technique, our model predicted initial bone formation across the craniotomy by 36 months of age. This is in line with observational studies of the same technique performed at a similar age^52,53^. A minor vertex bulging was evident by 36 months across the anterior-fontanel region. Such characteristics have been linked to ossification delays reported by Marucci et al.,^54^, who investigated the causes of ‘copper beaten’ appearances in previously treated MSC patients. Although our predictions display bone formation at the craniotomy by 36 months of age, large patency was seen at 9 months, which has resulted in a characteristic vertex bulging.

### Contact pressure

Further to predicting morphological and ossification outcomes, this work highlights the changes in pressure across the, here, ICV. Our results highlight that the MSC technique perhaps constrains the growth of the ICV (as a whole) to a larger extent compared to the SAC techniques. This observation was most apparent by 36 months, where pressure was higher in isolated regions (Fig. 7). This prediction suggests that improved morphological outcomes, as seen in this work, may not correlate to unrestricted growth and thus, lower overall pressure. Whether this higher pressure has any neurofunctional impact on brain growth or not can not be commented based on our data at present and requires a much more detailed clinical investigation. Nonetheless, this study highlights the huge potentials of finite element methods in understanding the biomechanics of different management techniques have on brain growth.

### Limitations

Despite promising resemblances between the in silico and in vivo results reported in this study, our study has several limitations. (1) Our simulations establish a bone-craniotomy & bone-suture lining method of formation. In reality, it is known that the dura mater possesses osteogenic properties which promote spontaneous ‘islands’ of bone across large calvarial defects^55^. Such advanced complexities and factors of ossification were not modelled in this study while these can be considered in future studies. Further, the values determined for replicating bone stiffness changes (i.e. 100 and 125 MPa for each month) and constituting the stage of ‘closed’ for both sutures and craniotomy stated here is highly generic and may not represent the true changes in ossification postoperatively. (2) Our approach in predicting ICV pressure postoperatively aimed to compare the potential benefits between techniques and assess brain growth^56^. It has been suggested that different surgical techniques can result in different neuropsychological outcomes, but such relations are disputed^56,57^. If at all, surgical outcome relates to neuropsychological outcome, our presented method predicts relevant skull size measures such as length, width and ICV and also predicts a more dynamic parameter, pressure, in the form of contact pressure mapping. Therefore, the presented method provides not only predictions of the morphological outcome but introduces a parameter with potential direct physiological significance. If in the future, we manage to determine the impact of the different outcome parameters on neuropsychological outcomes, FE models will add considerable value to surgical planning.

## Conclusion

The current study is, to the best of our knowledge, the first comparative analysis in predicting various treatment outcomes for sagittal craniosynostosis using a FE approach. The discussed results show promising perspectives in accurately predicting post-operative morphology and characteristics seen in vivo and various reported scenarios. Further work aims to broaden the current number of techniques in this study and evaluated the biomechanical impact of these techniques accordingly.

## Supplementary Information

Supplementary Information.
