# Supplementary material for: Predicting and comparing three corrective techniques for sagittal craniosynostosis
Source: Sci Rep. 2021 Oct 27;11:21216. doi: 10.1038/s41598-021-00642-7 (PMC8551239; doi:10.1038/s41598-021-00642-7)
Supplement: Supplementary file 1 — Supplementary Information. [file 41598_2021_642_MOESM1_ESM.docx]

**Supplements**

**Table S1:** Summary of sensitivity tests performed on the 2 SAC model to investigate the effect of various input parameters on the spring opening outcomes. Parameters of elastic modulus, the incorporation of the ICV, and contact behavior were all investigated under 10 scenarios. Note, (1) applicable changes in parameters are highlighted for each scenario; (2) n/a indicates no change from the baseline model values.

| **Scenario #:** | **Structure:** | **Bone properties**  **(MPa):** | **Suture properties**  **(MPa):** | **Craniotomy properties**  **(MPa):** | **ICV properties**  **(MPa):** | **ICV**  **present:** | **surface-to-surface behaviour:** |
| --- | --- | --- | --- | --- | --- | --- | --- |
| Baseline model | Whole skull: | 421 | 30 | 0.3 | 10 | Yes | contact |
| 1 | Half skull: | n/a | n/a | n/a | n/a | No | none |
| 2 | Whole skull: | n/a | n/a | n/a | n/a | No | none |
| 3 | Whole skull: | n/a | n/a | 0.03 | n/a | No | none |
| 4 | Whole skull: | 41 | n/a | n/a | n/a | No | none |
| 5 | Whole skull: | n/a | 3 | n/a | n/a | No | none |
| 6 | Whole skull: | n/a | n/a | n/a | 1 | Yes | fixed |
| 7 | Whole skull: | 41 | 3 | n/a | n/a | No | none |
| 8 | Whole skull: | 41 | n/a | n/a | 1 | Yes | fixed |
| 9 | Whole skull: | n/a | n/a | n/a | n/a | Yes | fixed |
| 10 | Whole skull: | 41 | n/a | n/a | 1 | Yes | contact |

**Table S2:** Results of sensitivity tests performed on the 2 SAC model, summarising spring opening outcomes at release corresponding to the scenarios described in Table S1. The elastic modulus of the bone, craniotomy and ICV were found to have the most profound effect on the spring opening at release. Achieving the closest displacement seen *in vivo* (approximately 5mm – See [10,34]). A fixed contact interface was seen to reduce the opening.

| **Scenario #:** | **Anterior spring (mm):** | **Posterior spring (mm):** |
| --- | --- | --- |
| baseline model | 15.2 | 15.2 |
| 1 | 16.4 | 16.5 |
| 2 | 16.1 | 16.2 |
| 3 | 16.09 | 16.19 |
| 4 | 15.03 | 14.9 |
| 5 | 16.09 | 16.19 |
| 6 | 15.89 | 16.01 |
| 7 | 15.03 | 14.9 |
| 8 | 15.23 | 15.25 |
| 9 | 15.7 | 15.7 |
| 10 | 18.58 | 19.15 |

**Table S3:** Sensitivity of craniotomy width and healing rate carried out based on the spring-assisted cranioplasty (SAC) with two springs and modified strip craniotomy (MSC) technique. Four scenarios were investigated here. Each scenario represented a variation of the craniotomy width (i.e. 5 vs. 50mm) and healing rate (i.e. 0.8mm vs. 10.8mm). Skull growth was modelled up to 36 months of age. The skull length, width, bone formation and contact pressure were all investigated between each scenario (see results corresponding to these tests in Figure S1).

| **Scenario number** | **Craniotomy formation rate (mm/month)** | **Craniotomy width (mm)** | **Surgical technique** |
| --- | --- | --- | --- |
| 1 | 0.8 | 5 | SAC |
| 2 | 0.8 | 50 | MSC |
| 3 | 10.8 | 5 | SAC |
| 4 | 10.8 | 50 | MSC |

**
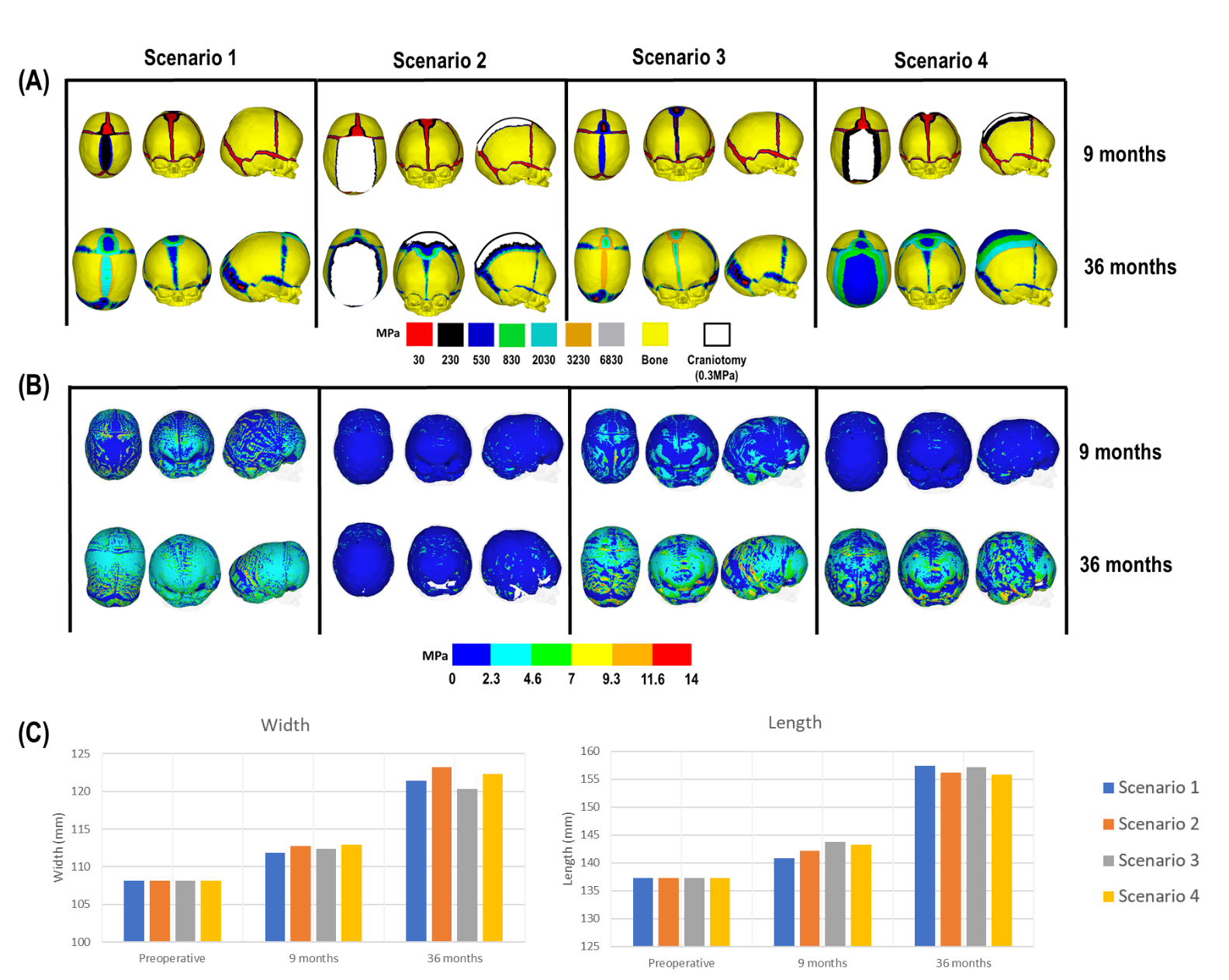
Figure S1:** The predicted outcomes of the sensitivity of craniotomy width and healing rate. Showing the level of bone formation (A), levels of contact pressure (B) and the cephalometric measurements at 76 months of age (C). The craniotomy within the scenario 3 was seen to close by 2 months postoperative until the formation rate of 10.8 mm vs. 5 months under scenario 1. Scenario 2 was seen to not achieve closure by 36 months under a formation rate of 0.8 mm. However, scenario 4 (10.8 mm) was seen to close by 12 months postoperative, achieving the closest match to *in vivo* estimations for achieving full healing.

**Table S4:** Sensitivity of 2 SAC spring forces: Spring opening and residual force outcomes. 8N, 9N and 10N of force across the springs from release to 9 months were investigated by increasing the spring stiffness (i.e. 0.094N/mm, 0.105N/mm and 0.117N/mm, respectively). Residual forces and opening distance were seen to increase by 0.9-1.0 N and 4-5 mm, respectively, for every newton of increased force.

| **Anterior spring:** | | | | **Posterior spring:** | | |
| --- | --- | --- | --- | --- | --- | --- |
|  | **8N springs** | **9N springs** | **10N springs** | **8N springs** | **9N springs** | **10N springs** |
|  | | | | | | |
| **Opening distance [mm]:** |  | | | | | |
| 3 months – insertion: | 15.1 | 15.1 | 15.1 | 15.0 | 15.0 | 15.0 |
| Release: | 18.5 | 18.9 | 19.3 | 19.1 | 19.6 | 20.1 |
| 6 months: | 28.6 | 29.0 | 29.4 | 28.8 | 29.2 | 29.8 |
| 9 months – removal: | 38.7 | 39.1 | 39.6 | 38.0 | 38.4 | 39.9 |
|  | | | | | | |
| **Residual forces**  **[N]:** |  | | | | | |
| 3 months – insertion: | 8 | 9 | 10 | 8 | 9 | 10 |
| Release: | 7.6 | 8.6 | 9.5 | 7.5 | 8.5 | 9.5 |
| 6 months: | 6.7 | 7.6 | 8.6 | 6.6 | 7.6 | 8.5 |
| 9 months – removal: | 5.7 | 6.7 | 7.6 | 5.8 | 6.7 | 7.7 |

**Table S5:** Sensitivity of 2 SAC spring forces: Cephalometric outcomes. Morphology was seen to have little change across all spring forces investigated.

|  | **Release:** | | | **6 months:** | | | **9 months - removal:** | | |
| --- | --- | --- | --- | --- | --- | --- | --- | --- | --- |
|  | **8N springs** | **9N springs** | **10N springs** | **8N springs** | **9N springs** | **10N springs** | **8N springs** | **9N springs** | **10N springs** |
| **Length [mm]:** | 135.8 | 135.9 | 136.6 | 138.0 | 137.2 | 138.1 | 139.7 | 139.2 | 139.8 |
| **Width [mm]:** | 106.9 | 107.5 | 107.3 | 109.8 | 109.1 | 108.8 | 114.2 | 114.8 | 110.7 |
| **Cephalic index:** | 78.4 | 79.0 | 78.5 | 79.8 | 79.5 | 78.8 | 81.7 | 82.4 | 79.2 |
| **Circumference [mm]:** | 375.6 | 376.2 | 376.8 | 389.4 | 397.3 | 399.0 | 422.2 | 400.6 | 385.6 |

**Table S6:** *In vitro* measurements of spring forces provided from the Sahlgrenska University Hospital (Gothenburg, Sweden). 9 devices were used in estimating the change in forces and length as crimping was performed. Initial length was measured at a mean of 100.3 mm, producing a force of 8.3 N when crimped to 15 mm (85 mm displacement). Length and forces were also examined post-crimping (i.e. measuring the plastic deformation of spring and resulting impact on forces), to which no change was seen in the mean force output (8.3 N) but reduced the mean leg-to-leg length of the spring (93.4 mm).

| Spring #: | Leg-to-leg initial length (mm): | Initial force when crimped to 15 mm (N): | Leg-to-leg length post- crimping (mm): | Secondary force at post-crimping again to 15mm- (N): |
| --- | --- | --- | --- | --- |
| 1 | 101 | 8 | 91 | 8 |
| 2 | 96 | 8.4 | 90 | 8.3 |
| 3 | 103 | 8.4 | 96 | 8.4 |
| 4 | 98 | 8.7 | 91 | 8.7 |
| 5 | 102 | 8.3 | 96 | 8.3 |
| 6 | 100 | 8.5 | 95 | 8.5 |
| 7 | 104 | 8.3 | 97 | 8.3 |
| 8 | 98 | 8.8 | 92 | 8.8 |
| 9 | 101 | 8.1 | 93 | 7.8 |
